# RNA interference-mediated *FANCF* silencing sensitizes OVCAR3 ovarian cancer cells to adriamycin through increased adriamycin-induced apoptosis dependent on JNK activation

**DOI:** 10.3892/or.2013.2295

**Published:** 2013-02-21

**Authors:** MIAO HE, HAI-GANG SUN, JUN-YING HAO, YAN-LIN LI, JIAN-KUN YU, YUAN-YUAN YAN, LIN ZHAO, NA LI, YAN WANG, XUE-FENG BAI, ZHAO-JIN YU, ZHI-HONG ZHENG, XIAO-YI MI, EN-HUA WANG, MIN-JIE WEI

**Affiliations:** 1 Department of Pharmacology, China Medical University, Heping, Shenyang, Liaoning 110001, P.R. China; 2 Institute of Pathology and Pathophysiology, China Medical University, Heping, Shenyang, Liaoning 110001, P.R. China

**Keywords:** ovarian cancer, FANCF, adriamycin, sensitivity

## Abstract

In the present study, we downregulated FANCF expression by small interfering RNA (siRNA) in OVCAR ovarian cancer cells to address the effects of decreased FANCF expression on the function of the Fanconi anemia (FA)/breast cancer susceptibility gene (BRCA) pathway. Furthermore, we investigated whether this method increases the sensitivity of OVCAR3 cells to adriamycin (ADM) and the possible mechanism(s). We found that silencing of *FANCF* inactivated the FA/BRCA pathway by decreasing the monoubiquitination and focus formation of FANCD2 and reduced the function of the FA/BRCA pathway, resulting in the inhibition of cell proliferation, increased cell apoptosis and DNA damage in OVCAR3 cells. Moreover, we observed that silencing of *FANCF* enhanced the antiproliferative effect of ADM in OVCAR3 cells and increased ADM intracellular accumulation consequently sensitizing OVCAR3 cells to ADM. Furthermore, silencing of *FANCF* increased cell apoptosis of OVCAR3 cells which was caused by decreased mitochondrial membrane potential (MMP)-induced DNA damage, activated Jun N-terminal kinase (JNK), increased release of cytochrome *c*, increased expression of cleaved caspase-3 and poly(ADP-ribose) polymerase (PARP) dependent on JNK activation following treatment of ADM. Collectively, we confirm that silencing of *FANCF* sensitizes OVCAR3 ovarian cancer cells to ADM, suggesting that FANCF may serve as a potential target for therapeutic strategies in the treatment of ovarian cancer.

## Introduction

Ovarian cancer is the second more prevalent gynecologic cancer and the fourth most common cause of death due to cancer among women (1,2). The standard treatment for ovarian cancer is surgical intervention followed by combination chemotherapy. Emerging data suggest that ovarian cancer cells are initially sensitive to chemotherapeutic drugs due to their genomic instability, and exhibit a good initial response. However, acquired resistance has become the most significant clinical problem and is a main obstacle to successful therapy for ovarian cancer (2–4).

To date, considering DNA repair pathways as the starting point with which to study tumorigenesis and resistance with complicated causes has shown promise (5,6). The Fanconi anemia (FA)/breast cancer susceptibility gene (BRCA) pathway, a DNA damage repair pathway, mediates proliferation, the cell cycle, apoptosis and invasiveness of tumor cells (7). FANCF, as an adaptor protein among 14 FA complementation (FANC) groups (FA-A, -B, -C, -D1, -D2, -E, -F, -G, -I, -J, -L, -M, -N and -P) and one FA-like complementation group (FA-O), is critically involved in regulating the function of the FA/BRCA pathway by maintaining the stability of the FA core complex and ubiquitin activation (monoubiquitination) of the FANCD2 protein (7–9). Epigenetic silencing of FANCF, such as methylation-induced inactivation of *FANCF*, plays an important role in the occurrence of several types of cancer including ovarian cancer via disruption of the FA/BRCA pathway (10–12). The disruption of the FA/BRCA pathway may prevent acquired resistance to DNA cross-linking agents and improve outcomes for cancer treatment. It has been reported that silencing of *FANCF* in resistant myeloma cells with small interfering RNA (siRNA) reversed resistance to melphalan (13). Taniguchi *et al*(10) found that FANCF demethylation resulted in cisplatin (CDDP) resistance in ovarian cancer cells. The FA pathway has also been reported to be critical in mediating cellular resistance to temozolomide (TMZ) and 1,3-bis[2-chloroethyl]-1-nitroso-urea (BCNU) (14). Thus, the FA/BRCA pathway, via FANCF, may represent a new target for preventing drug resistance and improving cancer treatment.

Adriamycin (ADM) remains the second-line agent for the treatment of patients with recurrent ovarian cancer after first-line platinum-based chemotherapy (15,16). However, the therapeutic effect of ADM has been significantly influenced by the development of resistance in cancer cells during treatment (17). Thus, it is necessary to find new strategies to improve the efficacy of chemotherapeutic agents and sensitize resistant cancer cells.

Here, we downregulated expression of FANCF by siRNA in an OVCAR ovarian cancer cell line and evaluated the effects of decreased FANCF expression on the function of the FA/BRCA pathway in OVCAR cells and their chemosensitivity to ADM. The results showed that downregulation of FANCF expression inhibited the function of the FA pathway in OVCAR cells and enhanced their susceptibility to ADM. It was also demonstrated that the enhanced sensitivity to ADM was associated with induction of apoptosis dependent on Jun N-terminal kinase (JNK) activation. Thus, interference of FANCF expression may be a new approach to improve chemosensitivity in the treatment for ovarian cancer.

## Materials and methods

### Cell culture

The human OVCAR ovarian cancer cell line was obtained from the American Type Culture Collection. Cells were maintained in RPMI-1640 (Invitrogen, Carlsbad, CA, USA) supplemented with 10% fetal bovine serum (FBS), 100 U/ml penicillin and 100 mg/ml streptomycin in a humidified atmosphere with 5% CO_2_ at 37°C.

### Antibodies and reagents

Antibodies against JNK, phospho-JNK, extracellular signal-regulated kinase (ERK), phospho-ERK, p38, phospho-p38 and β-actin were from Cell Signaling Technology (Beverly, MA, USA). Antibodies against FANCF, FANCD2, cytochrome *c* (cyt-*c*), cleaved-caspase-3 and cleaved-poly(ADP-ribose) polymerase (PARP) were from Abcam, Inc. (Cambridge, MA, USA). ADM, Annexin V, propidium iodide (PI), low melting point (LMP) and normal melting point (NMP) agarose, and 3-(4,5-dimethylthiazol-2-yl)-2,5-diphenyltetrazolium bromide (MTT) were purchased from Sigma Chemical Co. (St. Louis, MO, USA). The JNK inhibitor SP600125 was from Calbiochem-Merck (Darmstadt, Germany).

### Design of the siRNA targeting the FANCF gene and construction of the FANCF shRNA expression vector

According to the siRNA design guidelines, the RNA interference (RNAi) target sequence to the *FANCF* gene was designed (GeneBank accession no. NM022725.3) as follows: forward, 5′-GATCCGCTTCCTGAAGGTGATAGCGTTCAAGAGAC GCTATCACCTTCAGGAAGTTTTTTGGAAA-3′ and reverse, 5′-AGCTTTTCCAAAAAACTTCCTGAAGGTGAT AGCGTCTCTTGAACGCTATCACCTTCAGGAAGCG-3′.

FANCF small hairpin siRNA sequences were synthesized, annealed, and cloned into the pSilencer™ 4.1-CMV vector to generate the expression vector expressing FANCF shRNA. After amplication using standard methods, the recombinant plasmid was extracted and comfirmed by sequencing, then used throughout this study. A scrambled shRNA with no significant homology to human gene sequences was used as a negative control to detect nonspecific effects.

### FANCF shRNA transient transfection

Cells were seeded into 6-well plates (3×10^5^ cells/well) or 100 mm dishes (2×10^6^ cells) and allowed to adhere for 24 h, then transfected with the pSilencer™ 4.1-CMV control shRNA vector (control shRNA) or pSilencer™ 4.1-CMV FANCF shRNA vector (FANCF shRNA) using Lipofectamine 2000 (Invitrogen) according to the manufacturer’s instructions. After 4 h, the culture medium was replaced with fresh media supplemented with 10% FBS, and the cells were harvested at 24 and 48 h after transfection and used for the functional assay. For the determination of cell proliferation, cell numbers of viable cells were measured using a cell counter after staining dead cells with trypan blue.

### Western blot analysis

Total protein extracts from cells were obtained using RIPA lysis buffer containing 50 mM Tris, pH 8.0, 150 mM NaCl, 1% NP-40, 0.5% sodium deoxycholate and 0.1% sodium dodecyl sulphate (SDS). Prior to cell lysis, 0.1% phenylmethyl sulfonylfluoride (PMSF) and 1% phosphatase inhibitor were added to the lysis buffer. After shaking for 20 min on ice, the complex was centrifuged at 12,000 × g for 10 min at 4°C. The supernatants were collected. Protein quantification was carried out using a BCA kit (Walterson Biotechnology, Inc., Beijing, China). Samples were boiled in the presence of sample buffer (20% glycerol, 4% sodium dodecyl sulfate, 10% β-mercaptoethanol, 0.05% bromophenol blue and 1.25 M Tris-HCl, pH 6.8; all were from Sigma). Thirty micrograms of proteins was separated by electrophoresis on a 10% SDS-polyacrylamide gel and transferred to PVDF membranes. Blocking was carried out with 5% milk in Tris-buffered saline with 0.1% Tween-20 for 2 h at room temperature. Then the blots were incubated overnight at 4°C with the appropriate dilution of primary antibodies. After washing with PBST, the blots were incubated for 1 h with horseradish peroxidase-conjugated anti-IgG antibody (Santa Cruz Biotechnology, Inc.). Immunocomplexes were visualized using enhanced chemiluminescence (ECL) detection reagents (Santa Cruz Biotechnology, Inc.). The results of the protein expression were quantitatively analyzed with FluorChem v2.0 software (Alpha Innotech Corp., USA). The density [integrated density value (IDV)] of each protein expression band was normalized using the corresponding β-actin density as an internal control.

### Immunodetection of FANCD2 foci

OVCAR ovarian cancer cells were plated on glass coverslips at 50% confluence, and 16 h later were exposed to *FANCF* shRNA or control shRNA. At 24 and 48 h following exposure, cells were washed with phosphate-buffered saline (PBS), permeabilized with ice-cold 0.5% Triton X-100 in PBS, and then fixed with 2% paraformaldehyde and blocked with 5% bovine serum albumin at room temperature. FANCD2 was detected by incubation with the anti-FANCD2 antibody (1:500) for 90 min at room temperature and then with goat anti-rabbit antibody Alexa Fluor 488 (1:1,000; Invitrogen). All slides were counterstained with DAPI and visualized by fluorescence microscopy. The experiment was carried out in triplicate.

### Cell viability assay

Cell viability was assessed by MTT assay. Cells were seeded in 96-well plates at a density of 1×10^4^ cells/well and allowed to grow in the growth medium for 24 h. Cells were transfected with control or *FANCF* shRNA for 24 h and then treated with different concentrations of ADM for 24 h. After drug treatment, cells were incubated with 5 mg/ml (10 μl) MTT for 4 h at 37°C, then the medium was replaced with 100 μl dimethylsulfoxide (DMSO) and vortexed for 10 min. The absorbance (A) was recorded at 492 nm using a microplate reader. IC_50_ values were calculated from three independent experiments.

### Flow cytometry

Flow cytometric analysis was performed on a FACSCalibur (Becton-Dickinson). Twenty four hours after plating 3×10^5^ cells/well in 6-well plates, cells were transfected with control shRNA or FANCF shRNA. Cells were collected for the following studies at 24 h after transfection. Determination of the percentage of apoptotic cells was carried out using fluorescence isothiocyanate (FITC)-conjugated Annexin V (Annexin V-FITC) and PI. Cells were collected by centrifugation and washed twice with cold PBS, and the cell pellet was resuspended in 250 μl Annexin V-binding buffer at a concentration of 1×10^6^ cells/ml. The suspension (100 μl) was incubated in the dark at room temperature for 15 min with a solution of Annexin V-FITC (2.5 μg/ml) and PI (5 μg/ml). After addition of 400 μl of binding buffer to each tube, cells were analyzed for apoptosis by flow cytometry.

For the measure of the fluorescence intensity of intracelullar ADM, ADM was added to cells at a final concentration of 0.1 μg/ml. The cells were incubated for 1, 12 and 24 h at 37°C in 5% CO_2_ in the darkness. After the influx step, the cells were washed with ice-cold PBS. The intracellular fluorescence intensity of ADM was analyzed according to the fluorescence of ADM by flow cytometry.

Determination of mitochondrial membrane potential was carried out using a 5,5′,6,6′-tetrachloro-1,1′,3,3′-tetraethyl-imidacarbocyanine iodide (JC-1) kit. Cells treated with or without ADM (0.1 μg/ml) for 24 h after transfection were incubated for 15 min at 37°C with mitochondrial membrane potential-sensitive fluorescent dye JC-1 (15 μg/ml) and used to assess changes in the mitochondrial membrane potential by confocal laser scanning microscopy.

### DNA fragmentation assays

The alkaline comet assay was performed according to the procedure of Singh *et al*(18,19) with modifications. A freshly prepared suspension of cells in 0.6% LMP agarose dissolved in PBS was spread onto microscope slides precoated with 1% NMP agarose, covered with coverslips and allowed to set on ice for 10 min. After removing the coverslips, the slides with cells were then lysed for 1.5 h at 4°C in a cold lysis buffer consisting of 2.5 M NaCl, 100 mM EDTA, 10 mM Tris (pH 10), and 1% Triton X-100 was added immediately before use. After lysis, the slides were placed into an electrophoresis tank, and the DNA was allowed to unwind for 40 min in the electrophoretic solution consisting of 300 mM NaOH and 1 mM EDTA (pH >13.0). Electrophoresis was conducted at 4°C (the temperature of the running buffer did not exceeded 12°C) for 25 min at 300 mA. The slides were then transferred to neutralization solution with 0.4 M Tris-HCl (pH 7.5) for 3×5 min washes, then stained with 2.5 mM PI and covered with coverslips. To prevent additional DNA damage, all the steps described above were conducted under dimmed light or in the dark. Five hundred randomly chosen cells/slide were scanned and analyzed automatically using Casp1.01 software. Mean tail lengths were calculated for ~400 cells.

### Statistical analysis

Data obtained are representative of the averages of at least three independent experiments. All data are presented as means ± SD and analyzed using the one-way ANOVA with post-hoc analysis. P<0.05 was considered to indicate a statistically significant result.

## Results

### Silencing of FANCF reduces the function of the FA/BRCA pathway in OVCAR3 ovarian cancer cells

**Figure 1:**
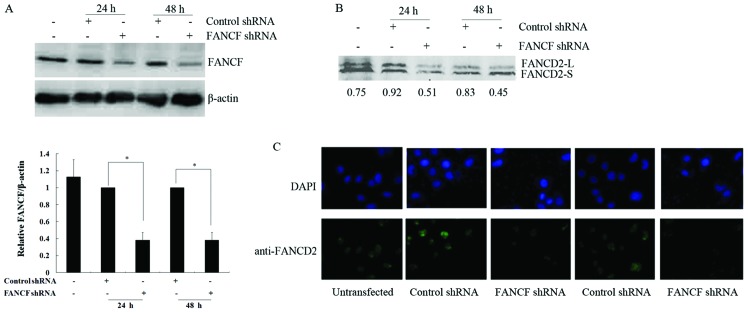
Inhibition of FANCF protein levels, FANCD2 ubiquitination and focus formation by *FANCF* silencing in OVCAR3 ovarian cancer cells. (A) Representative image of a western blot showing changes in FANCF protein expression in OVCAR3 cells at 24 and 48 h after transfection with *FANCF* shRNA. Protein quantification was carried out by densitometric analysis. Quantified FANCF protein was relative to the internal control β-actin. The relative FANCF/β-actin in control shRNA-transfected cells was considered equal to 1 in each experiment. Data are presented as the mean ± SD of at least three independent experiments. ^*^P<0.05, compared with control shRNA-transfected cells. (B) Representative image of a western blot showing changes in the monoubiquitination of FANCD2 in OVCAR3 cells at 24 and 48 h after transfection with *FANCF* shRNA. The relative level of the monoubiquitinated FANCD2 (FANCD2-L) was normalized to non-ubiquitinated FANCD2 (FANCD2-S) and was expressed as the ratio of FANCD2-L/FANCD2-S as indicated at the bottom of the image. (C) FANCD2 focus formation was detected by immunofluorescence, and representative images are shown.

In the present study, shRNA was used to knockdown *FANCF* expression in OVCAR3 ovarian cancer cells. The expression of FANCF protein was evaluated by western blot analysis in OVCAR3 cells at 24 and 48 h after transfection with *FANCF* shRNA. FANCF protein levels decreased to 38.1±9.2 and 38.1±9.1% of the control at 24 and 48 h after transfection, respectively (P<0.05) (Fig. 1A). The results confirmed that FANCF expression was inhibited by transfection with *FANCF* shRNA, and there was no obvious difference in the results of *FANCF* silencing between the 24- and 48-h transfection.

The monoubiquitination of FANCD2 is a key step in activating the FA/BRCA pathway (20). Thus, in order to verify whether *FANCF* silencing alters the function of the FA/BRCA pathway, we detected the changes in FANCD2 monoubiquitination after *FANCF* shRNA transfection in OVCAR3 cells by western blot analysis. The result showed that the ratios of monoubiquitinated FANCD2 (FANCD2-L)/non-ubiquitinated FANCD2 (FANCD2-S) were obviously decreased at 24 h (0.51 vs. 0.92) and 48 h (0.45 vs. 0.83) after transfection, when compared with the negative control (Fig. 1B). The data indicated that *FANCF* silencing reduced the monoubiquitination of FANCD2. Furthermore, we also observed reduced FANCD2 foci in *FANCF* shRNA-transfected cells when compared with the negative control at 24 and 48 h after transfection by immunofluorescence (Fig. 1C). These results suggest that *FANCF* silencing induces the inactivation of the FA/BRCA pathway in OVCAR3 ovarian cancer cells.

**Figure 2:**
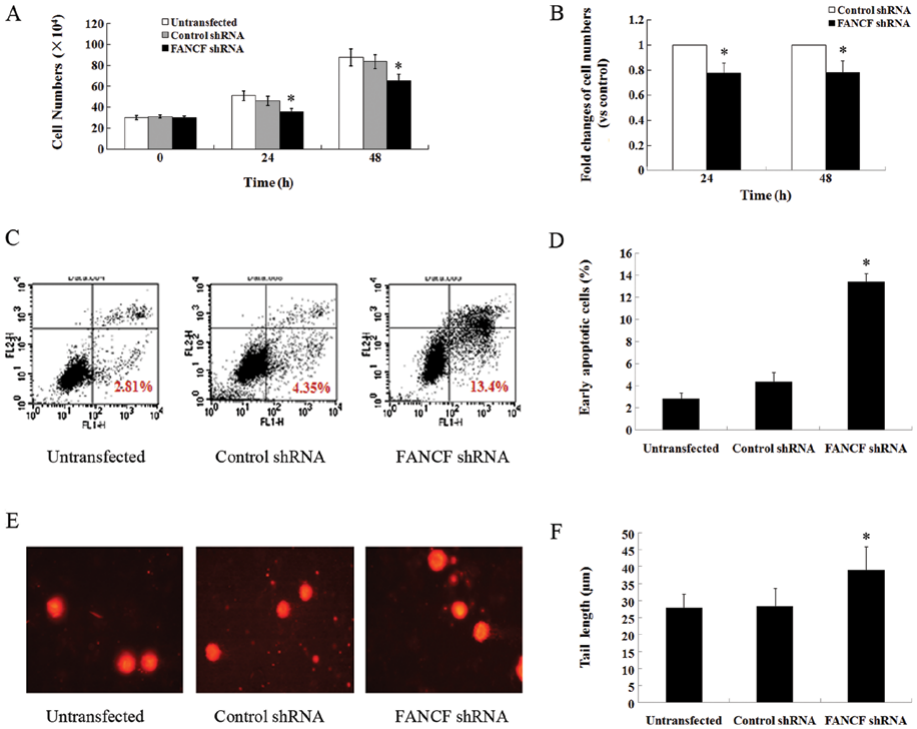
*FANCF* silencing inhibits proliferation, induces cell apoptosis and DNA damage in OVCAR3 ovarian cancer cells. (A) The numbers of viable cells were determined using a cell counter after staining dead cells with trypan blue at 24 and 48 h after transfection with either *FANCF* shRNA or control shRNA. (B) Quantitative analysis of the fold change in the total cell number of *FANCF* shRNA-transfected cells compared with shRNA-transfected cells. The cell number in the control shRNA-transfected cells was considered equal to 1 in each experiment. ^*^P<0.05, compared with control shRNA-transfected cells. (C) Apoptosis of OVCAR3 cells was measured by flow cytometry after staining with FITC-Annexin V/PI at 24 and 48 h after transfection with *FANCF* shRNA or control shRNA. Cells in the lower right quadrant represent early apoptotic cells with exposed phosphatidylserine (FITC-Annexin V-positive), but intact membrane (PI-negative). (D) Quantitative analysis of the percentage of early apoptotic cells in OVCAR3 cells. (E) Single-cell gel electrophoresis (comet assay) reveals detectable DNA damage in the form of DNA fragmentation visualized under a fluorescence microscope. (F) Quantification of tail lengths (μm) of the comet from 30 comets for each group. ^*^P<0.05, compared with control shRNA-transfected cells.

The main function of the FA/BRCA pathway is to mediate cell proliferation, apoptosis, and DNA damage repair (7). To further verify the blocked function of the FA/BRCA pathway by *FANCF* silencing in OVCAR3 cells, we first measured the effects of *FANCF* silencing on cell proliferation. *FANCF* shRNA decreased the total cell number to 77.6±8.5% of the control at 24 h and 78.3±9.2% of the control at 48 h after transfection in OVCAR3 cells (P<0.05) (Fig. 2A and B). Since treatment for different hours produced no obvious differences as revealed in the two above experiments, we chose 24 h as the transfection time in the subsequent experiments.

Flow cytometric analysis with Annexin V-FITC/PI staining was carried out to explore the effects of *FANCF* silencing on the apoptosis of OVCAR3 cells. The percentage of early apoptotic cells in the *FANCF* shRNA-transfected OVCAR3 cells was significantly higher (13.40±0.778%, P<0.05) than that in the control cells (4.352±0.843%) at 24 h after transfection (Fig. 2C and D; in the lower right quadrant of C). In addition, we explored the effects of *FANCF* silencing on cellular DNA damage by comet assay, a biomarker of apoptosis. The *FANCF* shRNA-transfected OVCAR3 cells were found to exhibit DNA damage in the form of fragmentation and longer tail length of the comet (39.1±6.79 μm, P<0.05) compared to the control cells (28.4±5.17 μm) (Fig. 2E and F). Taken together, these findings suggest that *FANCF* silencing reduces the function of the FA/BRCA pathway and inhibits proliferation, induces cell apoptosis and DNA damage in OVCAR3 cells.

### FANCF silencing sensitizes OVCAR3 ovarian cancer cells to ADM

**Figure 3:**
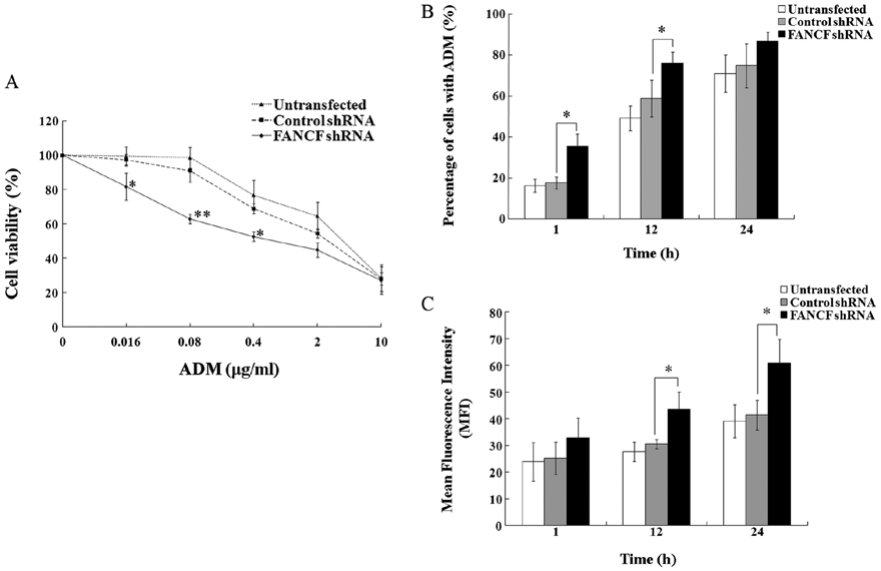
*FANCF* silencing sensitizes OVCAR3 cells to ADM. (A) Cell viability of OVCAR3 cells at different concentrations of ADM by MTT assay. (B) Percentage of OVCAR3 cells containing ADM following treatment of ADM (0.1 μg/ml) for 1, 12 and 24 h was analyzed by flow cytometry. (C) Mean fluorescence intensity (MFI) of intracellular ADM (0.1 μg/ml) at 1, 12 and 24 h in OVCAR3 cells was measured by flow cytometry. ^*^P<0.05, ^**^P<0.01, compared with control shRNA-transfected cells. ADM, adriamycin.

Since it was found that *FANCF* silencing inhibits the function of the FA/BRCA pathway in OVCAR3 cells, we aimed to ascertain whether *FANCF* silencing affects the antitumor effects of ADM on OVCAR3 cells. First, we evaluated the sensitivity of OVCAR3 cells to ADM by MTT assay. The dose-response curves showing the relationship between concentrations of ADM and cell viability revealed that cell viability was obviously decreased in the *FANCF*-silenced cells at ADM concentrations of 0.016, 0.08 and 0.4 μg/ml (P<0.05 or P<0.01) (Fig. 3A). The IC_50_ values (0.640±0.386 μg/ml) for ADM were markedly decreased after *FANCF* silencing, compared with the negative control (1.760±0.514 μg/ml, P<0.01). The results indicate that silencing of *FANCF* significantly enhances the antiproliferative effect of ADM in the OVCAR3 cells.

We next tested the effects of *FANCF* silencing on the intracellular accumulation of ADM in OVCAR3 cells by determining the percentage of cells containing ADM and the fluorescence intensity of intracellular ADM by flow cytometry. The percentages of cells containing ADM were obviously increased in the *FANCF*-silenced cells compared with the percentage in the control cells following treatment of ADM (0.1 μg/ml) for 1 h (35.52±5.93 vs. 17.58±3.01%, P<0.05) and 12 h (75.89±5.62 vs. 58.79±8.92%, P<0.05) (Fig. 3B). When compared with the control cells, the mean fluorescence intensity (MFI) was markedly increased in the *FANCF*-silenced cells following treatment of ADM (0.1 μg/ml) for 12 h (43.60±6.48 vs. 30.54±1.73, P<0.05) and 24 h (60.89±8.96 vs. 41.37±5.55, P<0.05) (Fig. 3C). These data further suggest that *FANCF* silencing sensitizes OVCAR3 ovarian cancer cells to ADM through increased ADM intracellular accumulation. Considering the greater accumulation of ADM following the treatment of ADM for 24 h, we treated the OVCAR3 cells with ADM for 24 h in the subsequent experiments.

### FANCF silencing increases ADM-induced apoptosis via JNK activation

**Figure 4:**
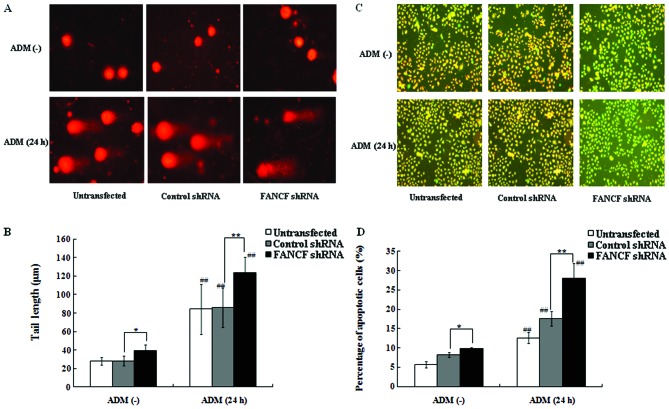
*FANCF* silencing increases ADM-induced DNA damage and apoptosis in OVCAR3 ovarian cancer cells. (A) Twenty-four hours after transfection, cells were treated with ADM (0.1 μg/ml) for 24 h and examined by single-cell gel electrophoresis (comet assay) showing detectable DNA damage in the form of DNA fragmentation visualized under a fluorescence microscope. (B) Quantification of tail lengths (μm) of the comet from 30 comets for each group. (C) Twenty-four hours after transfection, cells were treated with ADM (0.1 μg/ml) for 24 h and stained with JC-1 fluorescence dye, and changes in mitochondrial membrane potential (MMP) were examined by fluorescence microscopy. (D) Quantification of the percentage of apoptotic OVCAR3 cells. ^*^P<0.05, ^**^P<0.01, *FANCF* shRNA-transfected cells compared with control shRNA-transfected cells. ^##^P<0.01, the cells with treatment of ADM compared with the cells without ADM treatment. ADM, adriamycin.

Since *FANCF* silencing enhanced the antiproliferative effect of ADM in OVCAR3 cells, we hypothesized that *FANCF* silencing alters ADM-induced DNA damage, the main cytotoxic effect of ADM. Using comet assay again, we found that *FANCF*-silenced OVCAR3 cells and the control cells following treatment with ADM (0.1 μg/ml, 24 h) exhibited extensive DNA damage reflected by the tail length of the comet when compared with DNA damage in the cells without ADM treatment (P<0.01) (Fig. 4A and B). In addition, the *FANCF*-silenced cells were found to have increased DNA damage as evident from fragmentation and the longer tail length of the comet (123.46±17.35 μm) compared with the control cells (85.91±21.59 μm, P<0.01) following treatment of ADM (0.1 μg/ml, 24 h). These findings suggest that *FANCF* silencing increases the ADM-induced cellular DNA damage.

Decreased mitochondrial membrane potential (MMP) is a marker of early apoptosis and one of the reasons for DNA damage. We evaluated whether *FANCF* silencing increases the ADM-induced cellular DNA damage via decreased MMP in OVCAR3 cells by flow cytometry with JC-1 staining, a lipophilic and cationic dye. In normal cells, JC-1 concentrates in the mitochondrial matrix, where it forms red fluorescent aggregates (J-aggregates). In apoptotic cells with decreased MMP, JC-1 stays in the cytoplasm as monomers and fluoresces green (21). Without ADM treatment, there was an obvious increase in the percentage of apoptotic cells that emitted only green fluorescence, representing cells with depolarized mitochondrial membranes, in the *FANCF*-silenced cells (9.91±0.29%) when compared with the control cells (8.17±0.64%, P<0.05) (Fig. 4C and D). This result was consistent with the above findings by Annexin V-FITC/PI assay. Moreover, it was found that the treatment of ADM (0.1 μg/ml, 24 h) significantly increased the percentage of apoptotic cells, particularly in the *FANCF*-silenced cells (28.06±3.85%) when compared with the control cells (17.58±1.85%, P<0.01). These results indicate that *FANCF* silencing sensitizes OVCAR3 cells to ADM-induced apoptosis.

**Figure 5:**
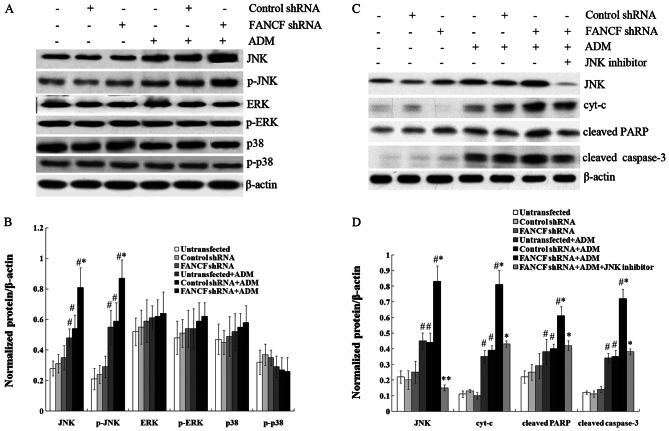
*FANCF* silencing increases ADM-induced apoptosis via JNK activation in OVCAR3 ovarian cancer cells. (A) Representative image of a western blot showing the ADM-induced changes in protein expression of JNK, p-JNK, ERK, p-ERK, p38 and p-p38 in OVCAR3 cells after *FANCF* silencing. (B) Protein quantification was carried out by densitometric analysis. Normalized proteins of JNK, p-JNK, ERK, p-ERK, p38 and p-p38 were relative to the internal control β-actin. (C) Representative image of a western blot indicating the ADM-induced changes in protein expression of JNK, cyt-c, cleaved caspase-3 and cleaved PARP in OVCAR3 cells after *FANCF* silencing and the treatment of JNK inhibitor SP600125. (D) Protein quantification was carried out by densitometric analysis. Normalized proteins of JNK, cyt-c, cleaved caspase-3 and cleaved PARP were relative to the internal control β-actin. ^*^P<0.05, ^**^P<0.01, *FANCF* shRNA-transfected cells compared with control shRNA-transfected cells, or *FANCF* shRNA-transfected cells with the treatment of JNK inhibitor compared with *FANCF* shRNA-transfected cells without treatment of JNK inhibitor. ^#^P<0.05, cells with treatment of ADM compared with cells without ADM treatment. ADM, adriamycin.

Activation of the mitogen-activated protein kinase (MAPK) pathway mediates ADM-induced cell apoptosis in multiple cancer cell lines (22). Using western blot analysis, we examined protein expression of genes related to the MAPK pathway, including JNK, ERK, and p38, in OVCAR3 cells following the treatment of ADM (0.1 μg/ml, 24 h) after *FANCF* silencing. These results showed that the expression of JNK and its phosphorylation level, but not the expression of ER and p38 proteins or their phosphorylation levels, was increased in OVCAR3 cells following the treatment of ADM (0.1 μg/ml, 24 h) compared with cells without ADM treatment. It was also found that only *FANCF* silencing without ADM treatment did not alter the expression of JNK, ERK, p38 proteins or their phosphorylation levels in OVCAR3 cells, indicating that *FANCF* silencing in OVCAR3 cells did not activate the MAPK pathway. However, *FANCF* silencing with ADM treatment (0.1 μg/ml, 24 h) notably increased the expression of JNK and its phosphorylation level compared with control cells (Fig. 5A and B). These results demonstrated that *FANCF* silencing increases ADM-induced JNK activation.

We also found that treatment with ADM (0.1 μg/ml, 24 h) significantly increased the expression of cyt-*c* from the release of mitochondria, cleaved caspase-3, and PARP in OVCAR3 cells by western blot analysis. Furthermore, *FANCF*-silenced OVCAR3 cells notably exhibited increased expression of these proteins induced by ADM. It was also shown that the increase in expression of these proteins was blocked by the JNK inhibitor SP600125 (Fig. 5C and D). These results indicate that FANCF silencing increases ADM-induced apoptosis via JNK activation.

## Discussion

FANCF protein is an important adaptor protein involved in the stabilizing component of a larger FA complex and maintains the biological functions of the FA/BRCA pathway (7–9). FANCD2 is expressed in normal human cells as two isoforms: FANCD2-S and FANCD2-L. DNA cross-linking agents, such as CDDP and mitomyclin C (MMC), and ionizing radiation (IR) can activate the conversion of FANCD2-S to FANCD2-L. The activated FANCD2 protein accumulates in nuclear foci in response to DNA-damaging agents and colocalizes with BRCA1. Central to the FA/BRCA pathway is the monoubiquitination of FANCD2, which connects upstream signaling with downstream enzymatic repair steps and activates the function of this pathway (23–25). Thus, the monoubiquitination and focus formation of FANCD2 are surrogate markers for FA/BRCA pathway activation (24,25). In the present study, we silenced the *FANCF* gene by RNA interference in OVCAR3 ovarian cancer cells and found decreased expression of FANCF protein, ratio of FANCD2-L/FANCD2-S and FANCD2 foci, which suggests inactivation of the FA/BRCA pathway by *FANCF* silencing in OVCAR3 ovarian cancer cells.

The main functions of the FA/BRCA pathway involve the cell cycle, DNA damage and repair, apoptosis, gene transcription and gene stability. Moreover, this pathway is necessary for cells to respond to DNA damage caused by IR, mitoxantrone (MX), CDDP and ADM (26,27). In the present study, we found that *FANCF* silencing inhibited proliferation, induced cell apoptosis and DNA damage in OVCAR3 cells, indicating that the function of the FA/BRCA pathway was blocked. Previous studies have reported that changes in the function of the FA/BRCA pathway affects the sensitivity of cancer cells to DNA-damaging agents (13,14). We also found that *FANCF* silencing increased the sensitivity of OVCAR3 ovarian cancer cells to ADM. To the best of our knowledge, this is the first evidence that blockage of the function of the FA/BRCA pathway by *FANCF* silencing in ovarian cancer cells increases the sensitivity of cancer cells to ADM. Although it has been reported that the TOV-21G cells absent of FANCF function with *FANCF* cDNA are resistant to MMC and CDDP (10), our study is the first to investigate the sensitivity of OVCAR3 ovarian cancer cells to another therapeutic drug ADM through loss of function of FANCF.

Following assessment of the cell viability at different concentrations of ADM, we found that *FANCF* silencing significantly decreased the IC_50_ values of ADM in OVCAR3 cells, suggesting the increased sensitivity of OVCAR3 cells to ADM. This enhanced antiproliferative effect of ADM in OVCAR3 cells by *FANCF* silencing was more obvious at concentrations from 0.08 to 0.4 μg/ml. Therefore, we selected the concentration of 0.1 μg/ml between the two concentrations to treat cells in subsequent experiments. Furthermore, we demonstrated that *FANCF* silencing increased the intracellular accumulation of ADM by measuring the percentage of cells containing ADM and the fluorescence intensity of intracellular ADM by flow cytometry and determined that it was one of the reasons for the increased sensitivity of OVCAR3 cells to ADM. Since the effect of the increased intracellular accumulation of ADM was more pronounced after treatment of ADM for 24 h, we chose this condition in subsequent experiments.

ADM is an active agent used for the treatment of patients with ovarian cancer (15,16). The main molecular target of ADM cytotoxicity is topoisomerase II that catalyzes a change in DNA topology via a concerted mechanism of transient DNA strand cleavage and religation. ADM can stabilize a transient DNA-topoisomerase II complex in which DNA strands are cut and covalently linked to the enzyme subunits. The stabilized complex results in DNA damage that is associated with the cytotoxic effect of ADM (28). In the present study, we found that ADM (0.1 μg/ml, 24 h) induced DNA damage of OVCAR3 cells through comet assay, and *FANCF* silencing increased the ADM-induced cellular DNA damage. One possible reason is that the function of the FA/BRCA pathway, in terms of DNA damage repair, was disrupted by *FANCF* interference in OVCAR3 cells, resulting in the decreased repair function of ADM-induced DNA damage.

Decreased MMP is one of the reasons for DNA damage. Many studies have shown that ADM accumulates in both the cellular nucleus and mitochondria and interferes with mitochondrial function and initiates the pathway of apoptosis by reducing MMP and releasing cyt-*c*(29–31). We observed that ADM induced a decrease in MMP and cell apoptosis, and *FANCF* silencing increased ADM-induced cell apoptosis in OVCAR3 cells. Activation of the MAPK pathway has been known to mediate ADM-induced cell apoptosis in multiple cancer cell lines (22). In the present study, treatment with ADM (0.1 μg/ml, 24 h) increased the expression of JNK and its phosphorylation level, induced the release of cyt-*c*, and increased the expression of cleaved caspase-3 and PARP dependent on JNK activation in OVCAR3 cells. These results were consistent with previous reports (32,33). Caspase-3 is an important executioner of apoptosis among the caspase family members. One of the substrates of caspase-3, PARP, a DNA repair enzyme, when cleaved, is inactivated and unable to repair DNA breaks or fragmentation, and plays a critical role in early apoptosis. The release of mitochondrial cyt-*c* is an important event in the apoptotic process and caspase-3 activation (34–36). Our data demonstrated that *FANCF* silencing increased the expression of ADM-induced cleaved caspase-3, cleaved PARP and cyt-*c* dependent on JNK activation, leading to cell apoptosis in OVCAR3 cells.

In conclusion, our findings demonstrated that *FANCF* silencing-induced dysfunction of the FA/BRCA pathway increased the sensitivity of the human ovarian cancer cell line, OVCAR, to ADM, by increased cell apoptosis dependent on JNK activation. We propose that FANCF may represent a novel target for enhancing the response of ovarian cancer cells to ADM, thus improving ovarian cancer treatment.
