# RNA interference-mediated FANCF silencing sensitizes OVCAR3 ovarian cancer cells to adriamycin through increased adriamycin-induced apoptosis dependent on JNK activation

**DOI:** 10.3892/or.2022.8327

**Published:** 2022-05-09

**Authors:** Miao He, Hai-Gang Sun, Jun-Ying Hao, Yan-Lin Li, Jian-Kun Yu, Yuan-Yuan Yan, Lin Zhao, Na Li, Yan Wang, Xue-Feng Bai, Zhao-Jin Yu, Zhi-Hong Zheng, Xiao-Yi Mi, En-Hua Wang, Min-Jie Wei

Oncol Rep 29: 1721–1729, 2013; DOI: 10.3892/or.2013.2295

After the publication of the article, an interested reader drew to the authors’ attention that there appeared to be a pair of overlapping data panels in Fig. 4C on p. 1726 [specifically, the ‘Untransfected’ and ‘Control shRNA’ data panels for the ADM (24 h) experiments]. The authors have consulted their original data, and have realized that this figure was inadvertently assembled incorrectly. Furthermore, they have noticed that Fig. 1 on p. 1724 also contained errors that arose during its assembly; essentially, several of the data panels in Fig. 1C, showing the detection of FANCD2 focus formation via immunofluorescence experiments, were selected inappropriately.

The corrected versions of Figs. 1 and 4, containing the corrected data panels for Figs. 1C and 4C respectively, are shown on the next page. Note that these errors did not affect the results or the conclusions reported in this work. The authors all agree to this Corrigendum, and are grateful to the Editor of *Oncology Reports* for allowing them to have the opportunity to correct these mistakes. Lastly, the authors apologize to the readership for any inconvenience these errors may have caused.

## Figures and Tables

**Figure 1.**
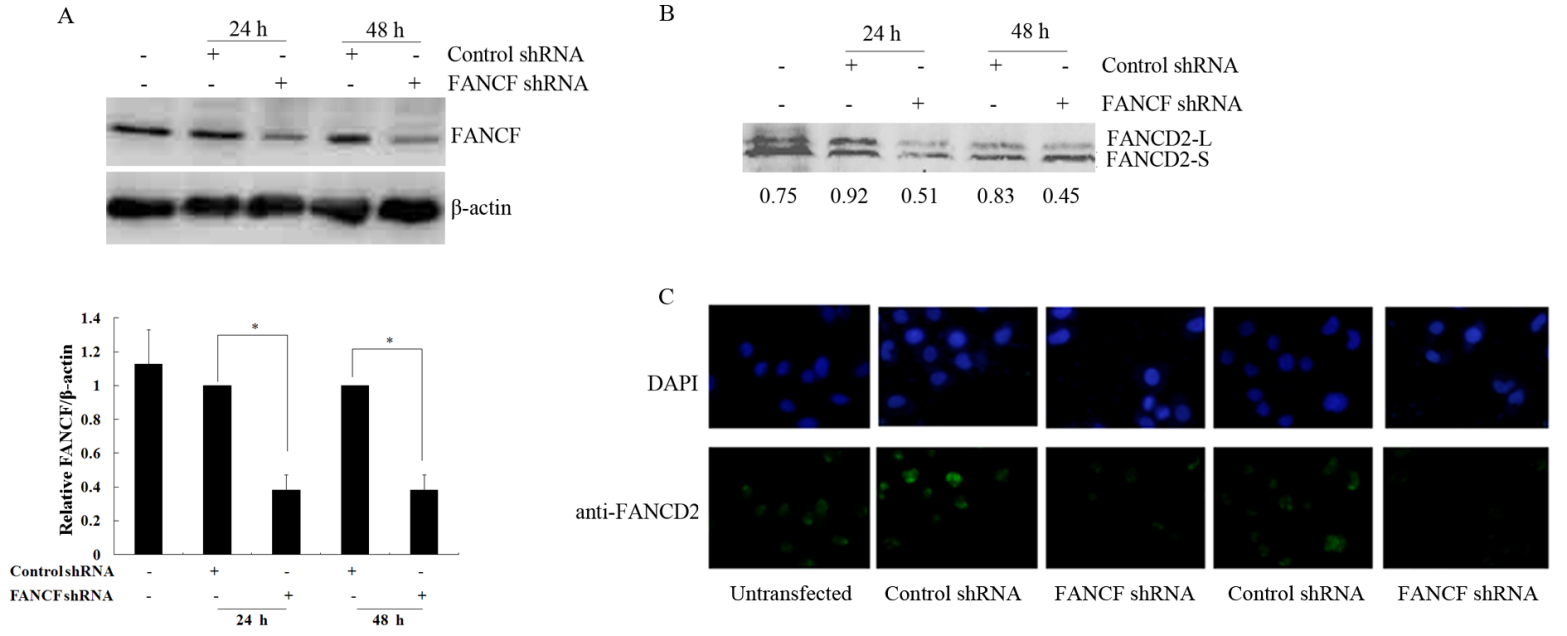
Inhibition of FANCF protein levels, FANCD2 ubiquitination and focus formation by FANCF silencing in OVCAR3 ovarian cancer cells. (A) Representative image of a western blot showing changes in FANCF protein expression in OVCAR3 cells at 24 and 48 h after transfection with FANCF shRNA. Protein quantification was carried out by densitometric analysis. Quantified FANCF protein was relative to the internal control β-actin. The relative FANCF/β-actin in control shRNA-transfected cells was considered equal to 1 in each experiment. Data are presented as the mean ± SD of at least three independent experiments. *P<0.05, compared with control shRNA-transfected cells. (B) Representative image of a western blot showing changes in the monoubiquitination of FANCD2 in OVCAR3 cells at 24 and 48 h after transfection with FANCF shRNA. The relative level of the monoubiquitinated FANCD2 (FANCD2-L) was normalized to non-ubiquitinated FANCD2 (FANCD2-S) and was expressed as the ratio of FANCD2-L/FANCD2-S as indicated at the bottom of the image. (C) FANCD2 focus formation was detected by immunofluorescence, and representative images are shown.

**Figure 4.**
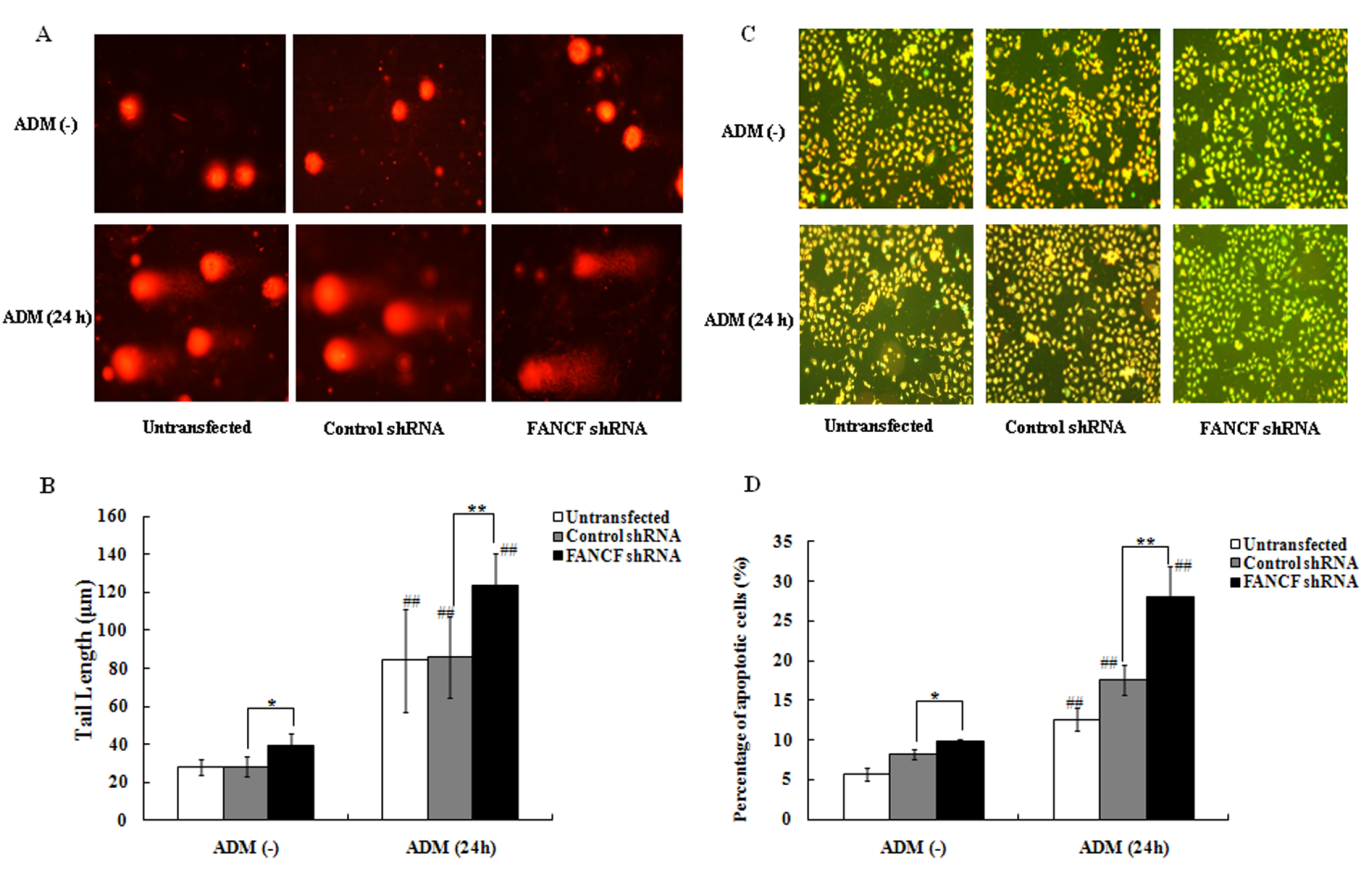
FANCF silencing increases ADM-induced DNA damage and apoptosis in OVCAR3 ovarian cancer cells. (A) Twenty-four hours after transfection, cells were treated with ADM (0.1 μg/ml) for 24 h and examined by single-cell gel electrophoresis (comet assay) showing detectable DNA damage in the form of DNA fragmentation visualized under a fluorescence microscope. (B) Quantification of tail lengths (μm) of the comet from 30 comets for each group. (C) Twenty-four hours after transfection, cells were treated with ADM (0.1 μg/ml) for 24 h and stained with JC-1 fluorescence dye, and changes in mitochondrial membrane potential (MMP) were examined by fluorescence microscopy. (D) Quantification of the percentage of apoptotic OVCAR3 cells. *P<0.05, **P<0.01, FANCF shRNA-transfected cells compared with control shRNA-transfected cells. ^##^P<0.01, the cells with treatment of ADM compared with the cells without ADM treatment. ADM, adriamycin.

